# Fibrolamellar hepatocellular carcinoma: a case report and gene analysis

**DOI:** 10.1186/s40792-023-01751-3

**Published:** 2023-09-20

**Authors:** Akira Watanabe, Norifumi Harimoto, Hideyuki Saito, Reika Kawabata-Iwakawa, Takaomi Seki, Ryo Muranushi, Kouki Hoshino, Kei Hagiwara, Norihiro Ishii, Mariko Tsukagoshi, Takamichi Igarashi, Kenichiro Araki, Hayato Ikota, Takashi Ishige, Koshi Mimori, Ken Shirabe

**Affiliations:** 1 Division of Hepatobiliary and Pancreatic Surgery, Department of General Surgical Science, Graduate School of Medicine, Gunma University, 3-39-22 Showa-Machi, Maebashi, Gunma 371-8511 Japan; 2 Department of Surgery, Kyushu University Beppu Hospital, Beppu, Japan; 3 Division of Integrated Oncology Research, Gunma University Initiative for Advanced Research, Gunma University, Maebashi, Japan; 4 Department of Diagnostic Pathology, Gunma University Hospital, Maebashi, Japan; 5 Department of Pediatrics, Graduate School of Medicine, Gunma University Maebashi, Maebashi, Japan

**Keywords:** Fibrolamellar hepatocellular carcinoma, Lymphatic metastasis, DNAJB1-PRKACA, Hepatic tumor, Lymph node metastasis, Gene mutation

## Abstract

**Background:**

Fibrolamellar hepatocellular carcinoma (HCC) (FL-HCC) is rare in Japan. FL-HCC develops in young patients with no history of cirrhosis and tends to manifest lymphatic metastasis with clinical features similar to those of HCC. We present a case of FL-HCC in a young male patient.

**Case presentation:**

A 14-year-old male patient underwent abdominal computed tomography (CT) to diagnose appendicitis, wherein a hepatic tumor was detected. Dynamic enhanced CT revealed a 35-mm solid tumor, which contrasted at the early phase of dynamic enhanced study of the right hepatic segments, with occlusion of the right portal vein. We performed right hepatectomy for these lesions. The patient experienced a single lymphatic recurrence on the hepatoduodenal ligament 12 months after the initial surgery. We performed lymphadenectomy for the recurrent tumor. We performed RNA sequencing (RNA-seq) and targeted DNA sequencing of the resected specimens (primary tumor, lymphatic metastasis, and normal liver). RNA-seq detected DNAJB1-PRKACA in both primary and metastatic lesions as previously reported. Furthermore, The Cancer Genome Atlas (TCGA) database was used to compare other gene expressions in this case with those of previously reported cases of FL-HCC and HCC in young patients. Principal component analysis of differentially expressed genes in the top 10% revealed that the gene expression in our case was similar to that of previous FL-HCC cases but was a different cluster from that in HCC cases in young patients. Mutational analysis did not detect any somatic mutations associated with carcinogenesis, including previously reported mutations (Kastenhuber et al. in Proc Natl Acad Sci USA 114: 13076–84, 2017).

**Conclusion:**

We encountered a case of FL-HCC, a rare hepatic tumor in an adolescent patient, and evaluated the genetic background. Our findings could contribute to the elucidation of the mechanisms underlying carcinogenesis and progression in patients with FL-HCC and thereby contribute to the development of new therapeutic strategies in the future that may improve patient prognosis.

## Background

Fibrolamellar hepatocellular carcinoma (HCC) (FL-HCC) accounts for 0.85% of all primary hepatic malignant tumors with an incidence of 0.02 per 100,000 cases in the United States. However, FL-HCC is rare in Japan and East Asia [1]. Few cases have been reported in Japan to date. Unlike “standard” HCC, FL-HCC typically develops in children or young adults without chronic liver disease. In addition, it is often detected when tumor size is relatively large because the initial stage is asymptomatic [2, 3]. The prognosis is similar to that of HCC. However, FL-HCC tends to easily result in lymph node metastasis [1]. The tumor cells possess eosinophilic granules in the cytoplasm, are arranged in a cord-like or sheet-like manner, and develop a layered structure along with the hyalinized connective tissue [4].

Recently, DNAJB1-PRKACA was reported to be a key factor in the development of FL-HCC [5]. However, the carcinogenic mechanism of this tumor remains unclear. Furthermore, no effective treatment method other than surgical intervention has been developed. Herein, we encountered FL-HCC in a young male patient. We analyzed the clinical course of treatment and the underlying gene mutation in this case.

## Case presentation

A 14-year-old male patient underwent abdominal computed tomography (CT) to assess appendicitis, wherein a 28-mm hepatic tumor was detected. The tumor increased in size during the 8-month follow-up period. The patient was referred to our department for complete examination because the tumor was considered to be possibly malignant. The patient had no history of viral hepatitis and no remarkable clinical history other than appendicitis. Blood test results showed carcinoembryonic antigen (1.8 ng/mL; [normal range, < 5.0 ng/mL]); carbohydrate antigen, 19–9 (4 U/mL [normal range, < 37 U/mL]); α-fetoprotein (1.5 ng/mL; [normal range, < 20 ng/mL]); α-fetoprotein isoform lectin affinity (< 0.5% [normal range, < 10%]); and protein induced by vitamin K absence or antagonist-II (36 mAU/mL; [normal range, < 40 mAU/mL).

**Fig. 1:**
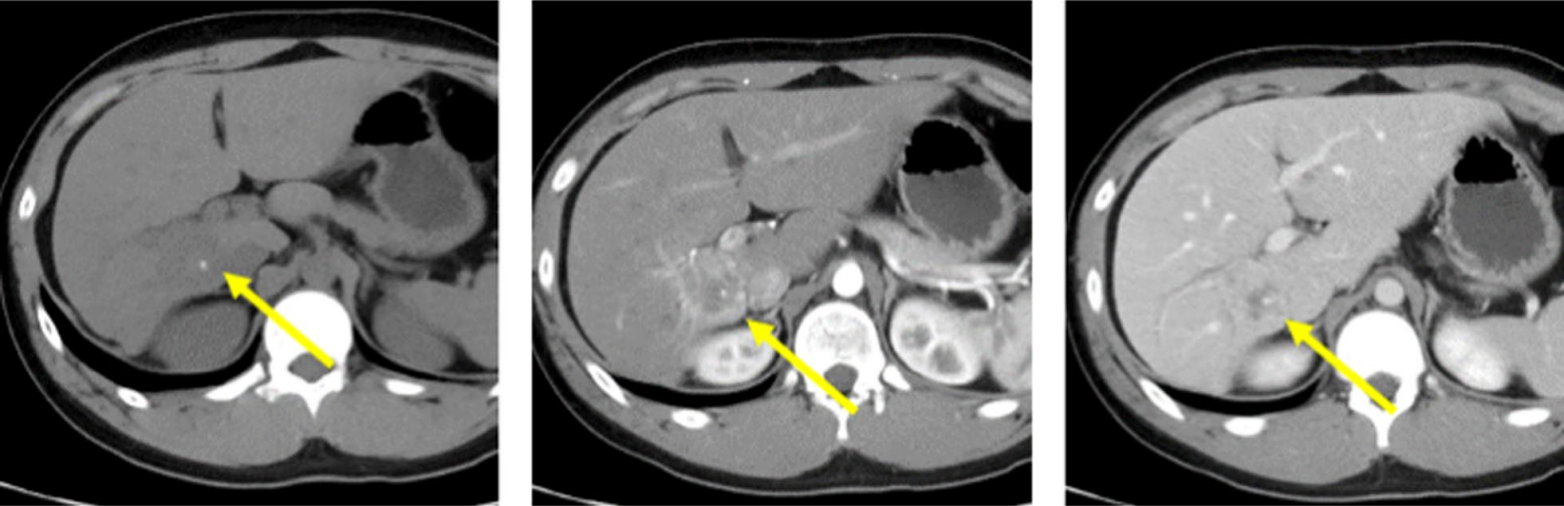
Abdominal CT showing a 35-mm solid tumor, which contrasts at the early phase of dynamic enhanced study of the hepatic segments 6, 7, and 8, with occlusion of the right portal vein and calcification in the tumor

**Fig. 2:**
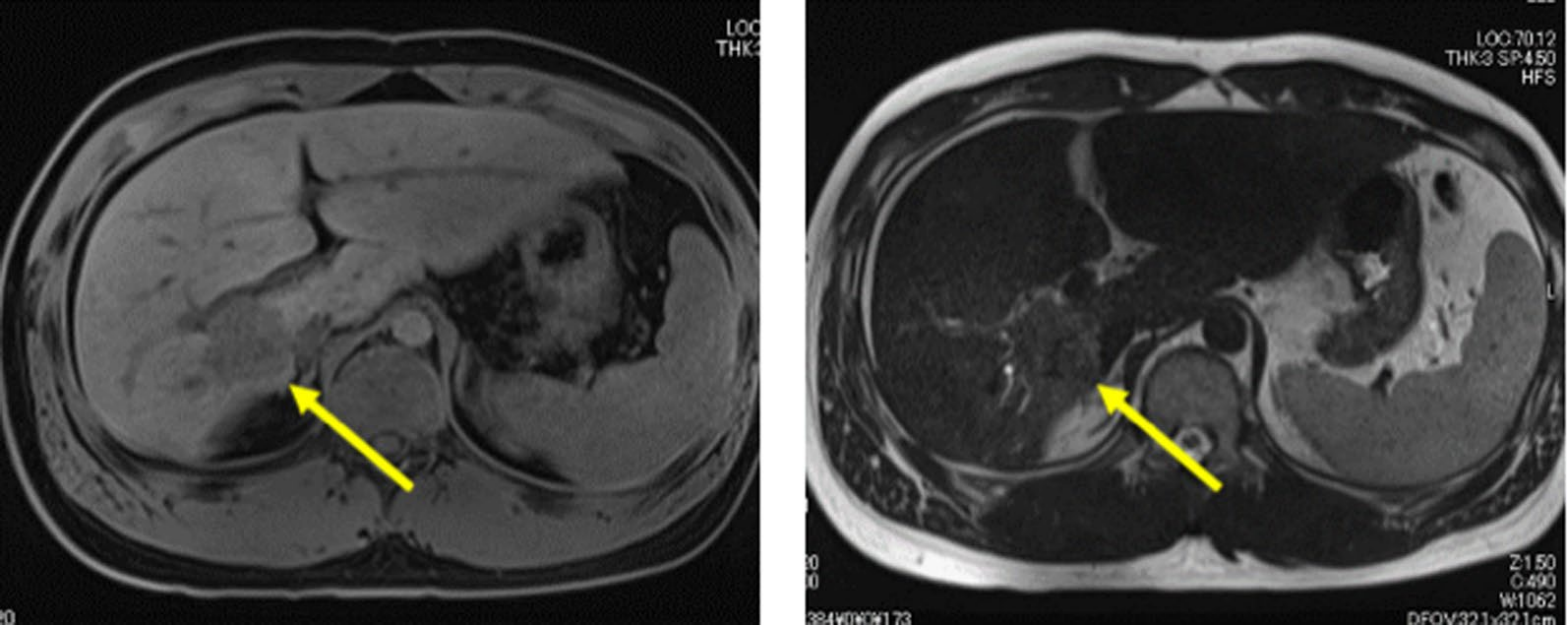
Magnetic resonance imaging (MRI) showing low signal tumor on T1-weighted image (T1WI), high signal marginal region on T2-weighted images (T2WI) and high signal marginal region on diffusion weighted image

Abdominal CT revealed a 35-mm solid tumor, which contrasted at the early phase of dynamic enhanced study of the hepatic segments 6, 7, and 8, with occlusion of the right portal vein and calcification in the tumor (Fig. 1). Magnetic resonance imaging (MRI) revealed a low signal on T1-weighted images (T1WI), a high signal marginal region on T2-weighted images (T2WI), and a high signal marginal region on diffusion weighted images (Fig. 2). The tumor manifested a central scar that displayed hypointensity on T1WI. ^18^F‑fluorodeoxyglucose (FDG) positron-emission tomography (FDG-PET) displayed FDG uptake (SUV max 2.4) at the tumor site. Based on these results, we diagnosed the patient with a malignant FL-HCC tumor. Therefore, we decided to perform surgical resection of the tumor.

**Fig. 3:**
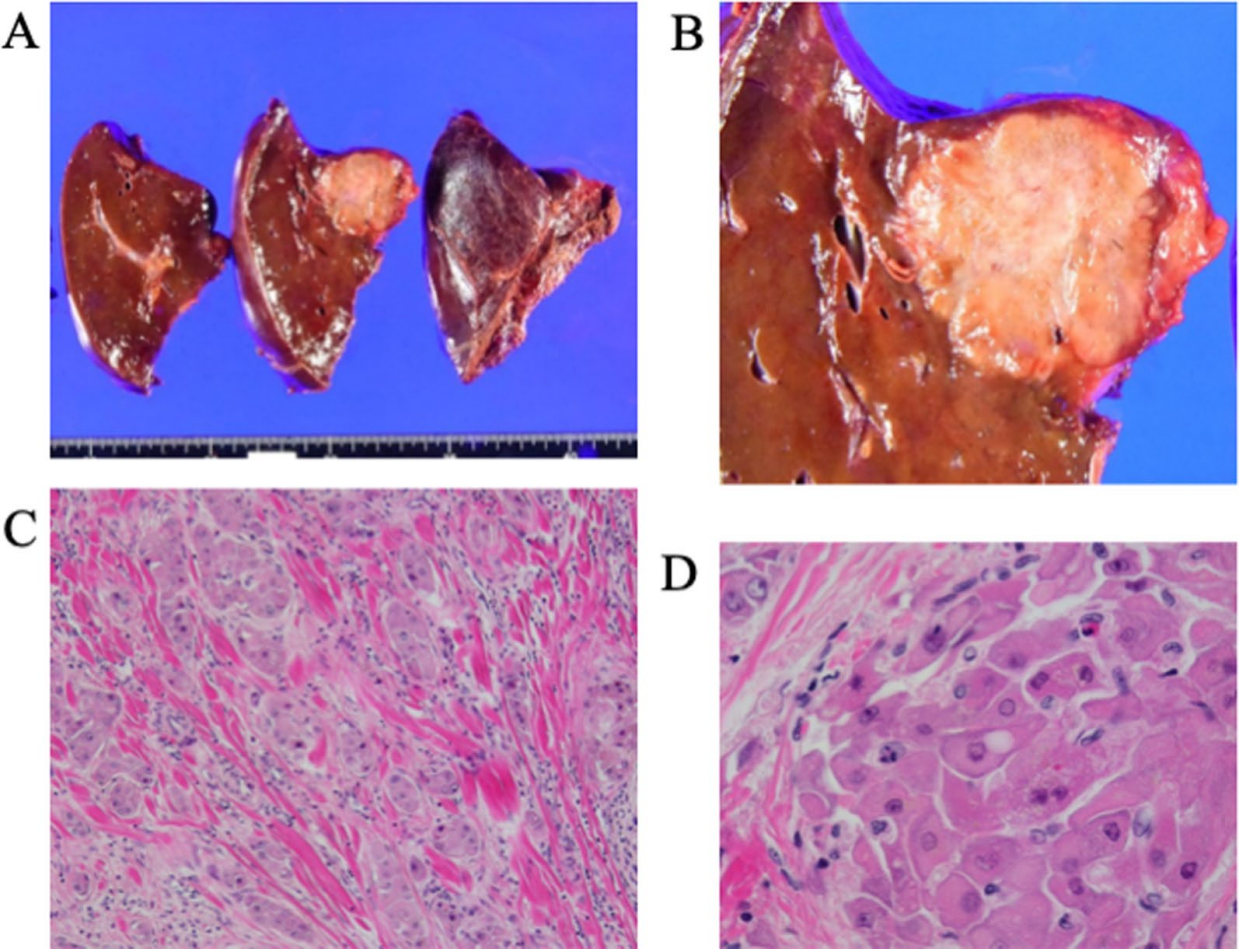
**a**, **b** Photograph of resected specimen. The tumor is a solitary nodule located on the right hepatic lobe. **c**, **d** Histopathological findings show that the central scar of the tumor has hyperplasia and fibrosis with hyaline calcification. The tumor cells possess enlarged nuclei with distinct nucleolus and eosinophilic granular cytoplasm. The tumor cells infiltrate, proliferate and divide into collagen fibers while forming a cord-like structure. **C** Hematoxylin–eosin staining, × 100. **D** Hematoxylin–eosin staining, × 400

The patient exhibited normal liver function (total bilirubin, 0.8 mg/dL; ICG-R15, 5.2%) and would retain sufficient residual liver volume (67.5%) after right hepatectomy. Therefore, we decided to perform right hepatectomy. The tumor excluded the right Glisson’s sheath: the right portal vein, right hepatic artery, and right hepatic duct were dissected individually, to enable the portal vein and common bile duct to be preserved. The enlarged lymph node (10 mm), which adhered to the tumor, was removed together with the right hepatic lobe. The postoperative course was favorable. The patient was discharged 9 days after surgery. The histopathological findings showed that the central scar of the tumor had hyperplasia and fibrosis with hyaline calcification. The tumor cells showed enlarged nuclei with a distinct nucleolus and eosinophilic granular cytoplasm. The tumor cells infiltrated, proliferated, and divided into the collagen fibers while forming a cord-like structure. The tumor also exhibited necrosis and calcification (Fig. 3). The resected lymph node showed tumor cell metastasis. Immunohistochemical analysis revealed positive results for hepatocyte, CK7, CK68, and AE1/AE3 (weakly positive), but CK19 did not present significant staining. Based on these findings, we diagnosed the patient with FL-HCC.

**Fig. 4:**
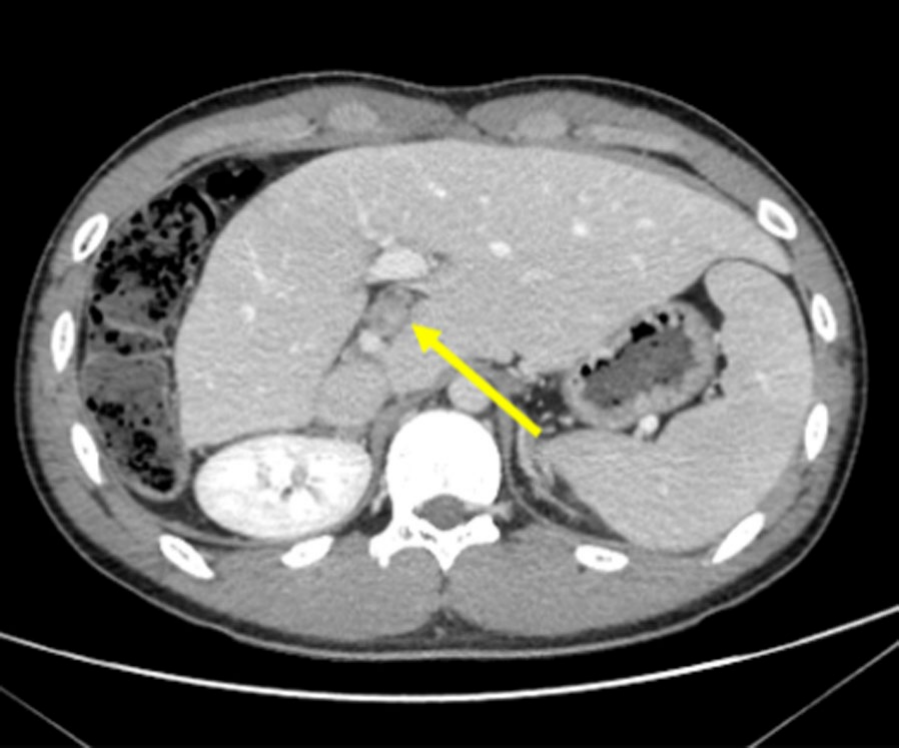
Abdominal CT shows a single lymphatic recurrence on the hepatoduodenal ligament (#12 lymph node) at 12 months after hepatectomy

The patient underwent enhanced CT or MRI every 3 months. He had single lymphatic recurrence on the hepatoduodenal ligament (#12 lymph node) at 12 months after hepatectomy (Fig. 4). We performed lymphadenectomy. However, the histopathological findings revealed that FL-HCC had recurred. The patient experienced no recurrence for 3 months following lymphadenectomy.

**Fig. 5:**
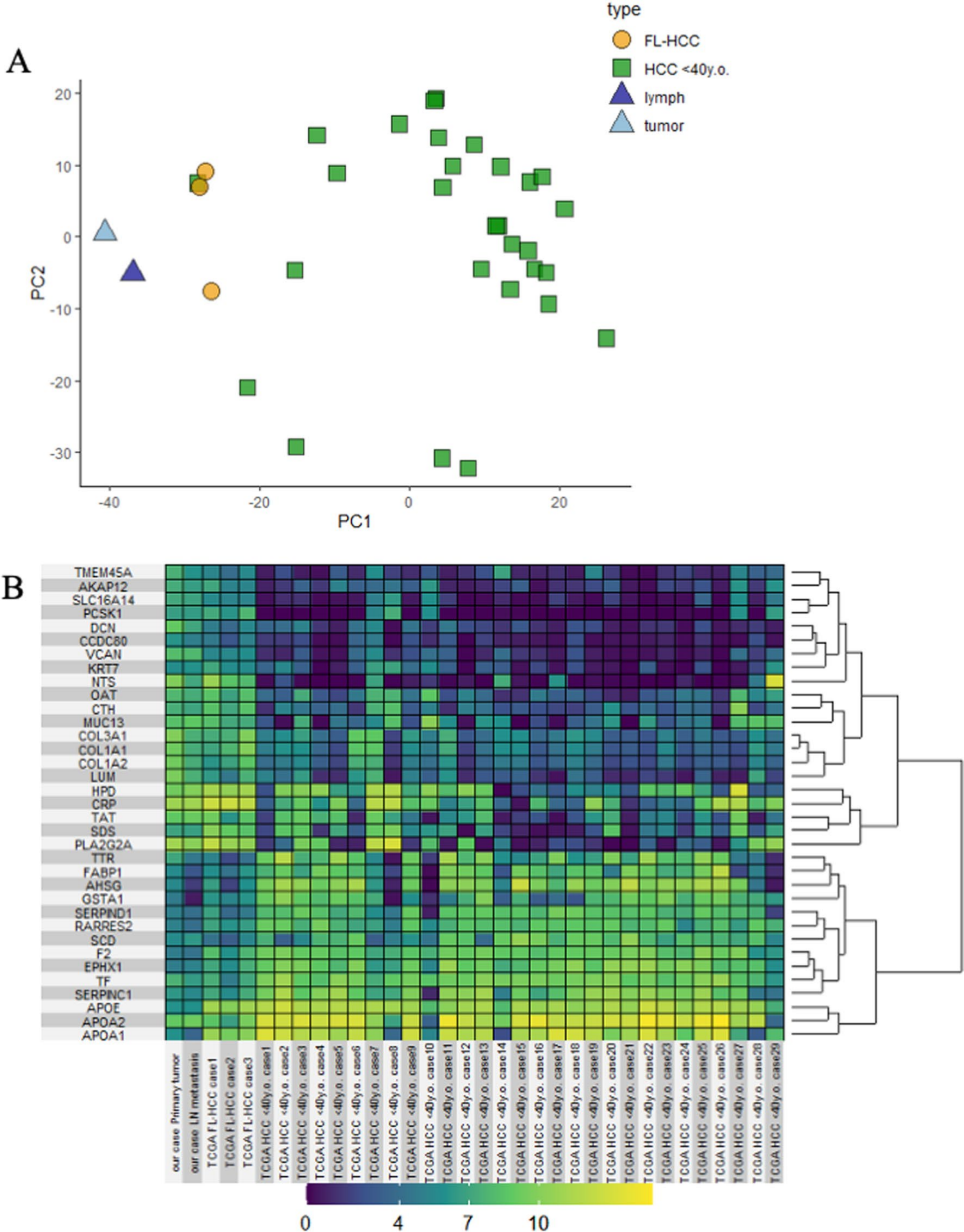
**a** Comparison of RNA-seq of the primary lesion (tumor: light blue triangle symbol) and the lymphatic metastatic lesion (lymph: light dark triangle symbol) in our case and in FL-HCC cases (*n* = 3: yellow circle symbol) and young (< 40 years) patients with HCC (*n* = 29: green squares) from TCGA database. Principal component analysis of differentially expressed genes in the top 10%; the gene expression of our case is similar to that of previous FL-HCC cases but is a different cluster from that of HCC cases in young patients. **b** Gene expression analysis of FL-HCC cases (our case and the previous 3 cases) and HCC cases in young patients. The heatmap shows that it clearly distinguished the top 21 and bottom 14 genes

We performed RNA-seq and targeted gene mutation analysis on formalin-fixed, paraffin-embedded specimens as previously described [6]. The number of input reads exceeded 50 million for all samples. Uniquely mapped reads were approximately 45 million (88%), 64 million (87%), and 49 million (91%) in the primary lesion, lymphatic metastatic lesion, and normal liver tissue, respectively. Fusion gene analysis was performed using DRAGEN RNA Pipeline v.3.5.7, identifying DNAJB1-PRKACA in both primary and metastatic lesions, but not in normal liver tissue, as reported in previous studies [1, 7–9]. We then compared the gene expression profiles of our case with those of previously reported cases of FL-HCC and HCC in young patients (< 40 years old) using The Cancer Genome Atlas (TCGA) database (http://xena.ucsc.edu/) to evaluate the features of gene expression. Figure 5 shows a comparison of the gene expression profiles of the primary and lymphatic metastatic lesions in our case, FL-HCC cases, and HCC cases in young patients from TCGA database. Figure 5a shows an analysis using the following procedure: we selected the top 10% of genes that were up-regulated in the tumor compared to those in normal liver tissue in 29 cases of HCC in young patients from the TCGA database: published by UCSC Xena of the University of California at Santa Cruz (http://xena.ucsc.edu/public). Based on the top 10% of genes, we created a list of gene expression levels for each sample (29 HCC cases in young patients from TCGA, 3 FL-HCC cases from TCGA, and our case). The RNA-seq data were RSEM normalized, and the count value (*x*) was calculated. The resulting log2(*x* + 1) was compared using the *t*-test. Principal component analysis (Fig. 5a) revealed that the gene expression profile in our case was similar to that of previous FL-HCC cases but was in a different cluster from that of HCC cases in young patients. The heatmap (Fig. 5b) shows the top 21 and bottom 14 genes with high differential expression in the analyzed FL-HCC cases (primary and lymphatic metastatic lesion in our case and the previous 3 FL-HCC cases) and HCC cases in young patients. The top 21 genes were selected based on the criteria that log2 (fold change) exceeds 3.5 and the bottom 14 genes were selected based on log2 (fold change) less than − 2.0. Table 1 summarizes the gene expressions for the 35 expression variants. According to gene set enrichment analysis, compared with HCC cases in young patients, FL-HCC cases exhibited alteration of gene expression related to epithelial to mesenchymal transition, hypoxia, and KRAS signal. Next, targeted 275 gene mutation analysis was performed using the Human Comprehensive Cancer QIAseq DNA Panel (QIAGEN) as previously reported [6]. The mean coverage depth and mean percentage of target coverage ≥ 20 × were 2766.7 and 97.2, respectively. Candidates for affectable mutation showed (1) amino acid change or variant on a splice site; (2) low minor allele frequency in Japanese/East Asian (< 0.01, HGVD2, DBexome20161214; https://www.hgvd.genome.med.kyoto-u.ac.jp/gnomAD exome EAS, v2.0.1); and (3) variant allele frequency > 0.05. Furthermore, multiple variants detected in single reads were removed, probably because of mapping errors owing to manual inspection of aligned reads using the Integrative Genomics Viewer (http://www.broadinstitute.org/igv/). Candidate somatic mutation was detected only in *PBRM1* (NM_018313: c.3603G > A: p.M1201I, VAF = 0.058) and not in lymph node metastases. In addition, this mutation was not confirmed by RNA-seq data. Therefore, it is likely that the mutant allele was not expressed. The previously reported *TERT, CTNNB1*, *APC*, *NOTCH1*, *EPHA5*, *BCOR*, and *CIC* mutations were not detected in our case [7].

**Table 1:** Comparison of gene expression between FL-HCC and young HCC

Gene Symbol	Gene Expression		*P*-value	Fold Change
	FL-HCC	HCC <40 y.o.		
	(*n*=5)	(*n*=29)		
*VCAN*	8.817148	1.616	< 0.001	7.2011
*CCDC80*	10.88519	4.140918	0.001	6.7442
*NTS*	6.840951	1.106541	< 0.001	5.7344
*PCSK1*	11.88166	6.896194	< 0.001	4.9854
*AKAP12*	6.534875	1.747636	< 0.001	4.7872
*TF*	6.011128	1.56449	< 0.001	4.4466
*COL1A2*	7.14167	2.956288	0.0014	4.1853
*COL1A1*	5.985437	2.101835	< 0.001	3.8836
*COL3A1*	7.628198	3.775831	0.001	3.8523
*CRP*	6.269426	2.442686	< 0.001	3.8267
*SLC16A14*	7.643811	3.823263	< 0.001	3.8205
*DCN*	8.26088	4.526932	< 0.001	3.7339
*EPHX1*	8.34207	4.626477	0.008	3.7155
*PLA2G2A*	7.922695	4.240189	0.0067	3.6825
*CTH*	7.742455	4.069396	0.0014	3.673
*TMEM45A*	8.165952	4.50216	0.0217	3.6637
*LUM*	6.046244	2.411356	0.0029	3.6348
*OAT*	5.642841	2.057272	0.001	3.5855
*F2*	8.380275	4.797066	< 0.001	3.5832
*RARRES2*	10.3543	6.812451	0.0159	3.5418
*SERPIND1*	4.699167	1.193037	< 0.001	3.5061
*KRT7*	5.719496	7.844187	0.0018	− 2.1246
*APOA2*	8.785837	10.92171	0.0252	− 2.1358
*MUC13*	4.904502	7.130208	0.0436	− 2.2257
*TAT*	4.591301	6.93159	0.0382	− 2.3402
*AHSG*	4.938774	7.312314	0.0187	− 2.3735
*HPD*	6.358531	8.736732	0.0217	− 2.3782
*SCD*	6.214376	8.63604	0.0014	− 2.4216
*SDS*	7.935027	10.36979	0.0382	− 2.4347
*SERPINC1*	6.672331	9.16285	< 0.001	− 2.4905
*APOE*	6.04407	8.785136	< 0.001	− 2.741
*TTR*	4.848222	7.63215	0.0023	− 2.7839
*GSTA1*	4.605411	7.427156	0.0252	− 2.8217
*APOA1*	8.581028	11.9013	0.0029	− 3.3202
*FABP1*	4.335924	8.726212	0.0115	− 4.3902

FL-HCC (*n* = 5: primary and lymphatic metastasis lesion in our case and previous 3 cases)

The gene names was referred the newest symbol of HGNC (HUGO Gene Nomenclature Committee)

The gene expression level was used by taking the FPKM value of mRNA as log2(*x* + 1)

P value was evaluated by Wilcoxon rank sum test. Fold Change is the expression ratio of FL-HCC to younger HCC

## Discussion

We encountered a surgical case of FL-HCC, which is rare in our country. We reviewed and analyzed the imaging, clinical, and genetic characteristics of this tumor.

Imaging findings revealed that FL-HCC exhibited low CT values with central scars (65–70%) and calcification (40–68%) reported as characteristic findings [10, 11]. Contrast-enhanced CT showed inhomogeneous staining in the arterial phase, with 50% isoattenuating, 36% hyperattenuating, and 16% hypoattenuating in the portal phase [12]. Portal vein thrombosis and biliary obstruction are rare manifestations of FL-HCC [10]. FL-HCC exhibits a low signal on T1WI and high signal on T2WI in MRI. It is a characteristic finding that the central scar displays hypointensity on both T1 and T2. Similar to the enhanced CT image, the MRI gadolinium-enhanced image shows a non-uniform contrast effect in the arterial phase and an isointense or hypointense contrast effect in the portal and parallel phases [12, 13]. Focal nodular hyperplasia is different from FL-HCC. Yamaguchi et al. suggested that FL-HCC manifested calcification on CT and low signal of the central scars on T2-enhanced MRI as a distinct difference in imaging findings [14]. In our case, the CT image of the tumor showed calcification and the central scar exhibited hypointensity on the T1WI MRI. FL-HCC is primarily detected in children as a large mass exceeding 7–8 cm [15]. In the present case, preoperative diagnosis was challenging because the size was smaller than in previous cases.

Surgical resection is the first-line treatment and promising curative methods for FL-HCC. Pinna et al. reported details of recurrence after hepatectomy for FL-HCC as follows: residual liver recurrence (44%), abdominal lymph node metastasis (33%), lung metastasis (29%), and mediastinal lymph node metastasis (10%) [16]. Lymphadenectomy is one of the treatments of lymph node recurrence, and long-term survival was reported for lymph node recurrence after lymphadenectomy [17, 18]. In our case, lymph node recurrence also occurred after hepatectomy, resulting in lymphadenectomy. Gummadi et al. stated that lymph node dissection may contribute to improved prognosis [19]. Based on these reports, we suggest that the surgical strategy should consider whether lymph node dissection should be performed in addition to hepatectomy. Radiation therapy has not exhibited a therapeutic effect [20]. Chemotherapy also has not been established. However, treatment with FOLFOX (oxaliplatin, 5-fluorouracil) and GEMOX (oxaliplatin, gemcitabine) has been reported [8, 9, 21]. Yamashita et al. reported the surgical outcomes and prognosis in 65 patients with FL-HCC. Recurrence free survival and overall survival were significantly affected by the number of tumors and vascular invasion but were not associated with lymphatic metastasis. Postoperative recurrence developed in 56 (86%) patients with FL-HCC, and repeat surgical resection was performed in 25 (45%) patients. The 5-year and median overall survival rates of 58% and 81 months, respectively, after resection in FL-HCC were similar to the 5-year and median overall survival rates after resection of non-cirrhotic HCC (67% and 137 months, respectively) [1].

Honeyman et al. first discovered a fusion gene ‘*DNAJB1-PRKACA*’ by performing whole-transcriptome and whole-genome sequencing of paired tumor and adjacent normal liver samples [5]. *DNAJB1* encodes a part of the heat shock 40 protein family, and *PRKACA* encodes adenosine 3′,5′-monophosphate (cAMP). DNAJB1-PRKACA was detected in FL-HCC but not in other HCC or normal hepatic tissue [22]. The induction of DNAJB1-PRKACA by genome engineering led to tumorigenesis of FL-HCC-like tumors in a mouse model [23]. Since DNAJB1-PRKACA may play a key role in FL-HCC development, DNAJB1-PRKACA could become a therapeutic target or prognostic marker.

The only somatic mutation detected was in *PBRM1*, which encodes a component of the SWI/SNF chromatin remodeling complex and is promising for poly (ADP-ribose) polymerase inhibitors in cases with loss-of-function mutations [24]. Both the Polyphen2_HDIV_pred and Polyphen2_HVAR_pred predictive scores of the detected variant were "Damaging", and according to the Catalogue Of Somatic Mutations In Cancer v96, identical mutations have been registered as somatic mutations in two cases of bladder cancer, without reporting functional effects. In addition, the biological significance of this mutation is unknown because it is detected only in primary tumors and its expression is not detected at the transcriptional level. Regarding PBRM1 mutations, Zimmer et al. reported therapeutic effects by targeting the DNA repair system [25]. In this study, PBRM1 knockdown in biliary tract cancer resulted in synergy with the drug efficacy of niraparib and olaparib in vitro. In clinical practice, the therapeutic efficacy of niraparib has been also reported in patients with advanced biliary tract cancer with PBRM1 mutant. PBRM1 may be a potential therapeutic target in FL-HCC cases in the future.

## Conclusion

FL-HCC is a rare malignant tumor that develops in young patients and often advanced at the time of discovery. Regarding treatment of FL-HCC, in addition to surgical resection, multidisciplinary treatment such as drug therapy could be important. Analysis of the genetic background of FL-HCC could provide important information for elucidating the mechanism of tumor progression. As a result, new therapeutic targets could be developed. Moreover, it is expected that these findings would contribute to improving treatment and prognosis in patients with FL-HCC in the future.

## Data Availability

The dataset supporting the conclusions of this article is included within the article.

## Abbreviations

CT: Computed tomography
FDG-PET: ^18^F‑fluorodeoxyglucose positron-emission tomography
FL-HCC: Fibrolamellar hepatocellular carcinoma
HCC: Hepatocellular carcinoma
MRI: Magnetic resonance imaging
RNA-seq: RNA sequencing
T1WI: T1-weighted images
TCGA: The Cancer Genome Atlas
